# On the Non-Existence of Sugar in the Blood of Persons Labouring under Diabetes Mellitus. In a Letter to Alexander Marcet, M. D. F. R. S. from William Hyde Wollaston, M. D. Sec. R. S.

**Published:** 1811-12

**Authors:** W. H. Wollaston


### *On the Non-existence of Sugar in the Blood of Persons labouring under Diabetes Mellitus. In a letter to* Alexander Marcet, *M. D. F. R. S. from* William Hyde Wollaston, *M. D. Sec. R. S.*

(Phil. Trans. P. I. 1811.)

Sir,

ALTHO' Dr. Rollo had been assisted in the chemical part of his inquiry by the well known talents of Mr. Cruickshank, it appears that they "had not been so fortunate as to obtain a sufficient quantity of serum for chemical experiment;"* and were unable fully to satisfy themselves by the taste or by other means which they could employ, concerning the existence or non-existence of sugar in the blood of persons labouring under diabetes; but nevertheless they were persuaded of its presence.

For the purpose of forming some judgment on this question, Mr. Cruickshank made trial of the quantities of oxalic acid that could be formed from serum or blood in their natural state, and from the same serum or blood after the addition of a certain proportion of sugar; and from the difference perceptible in these trials, he formed a probable conjecture respecting the presence or absence of sugar in the serum of diabetic persons.

This method, it is evident, is liable to a two-fold objection; first, that an access of other ingredients beside sugar will cause an increase of the quantity of oxalic acid formed, and secondly, that slight variations in the process for forming oxalic acid will unavoidably occasion differences in the result.

The method which I employed appears to me capable of detecting much smaller quantities of such an ingredient, for though it might not enable us to distinguish exactly the nature of any small quantity that may be discovered, still the mere question of absence or presence admits of determination with great precision.

For this purpose I investigated, in the first place, how the albuminous part of healthy serum could be most completely coagulated; and by what appearance the presence of sugar that had been added to it would be most easily discerned.

When heat alone had been employed for the coagulation of serum, to which water had been added, that which exsuded from it was still found to contain a portion of albumen dissolved in it, and if this were allowed to remain any saccharine

* Rollo on Diabetes, p 408.

matter matter which might be present would be disguised, and could not with certainty be detected.

I found, however, that this residuum of coagulable matter might be altogether prevented by the addition of a small quantity of dilute acid to the serum before coagulation.* To six drams of serum I added half a dram of muriatic acid previously diluted with one dram and a half of water, and immersed the phial containing them in boiling water during four minutes. The coagulation was thus rendered complete. In the course of a few hours a dram or more of water exudes from serum that has been so coagulated. If a drop of this water be evaporated, the salts which it contains are found to crystallise, so that the form of the crystals may be easily distinguished; they are principally common salt.

If any portion of saccharine matter has been added to the serum previous to coagulation, the crystallization of the salts is impeded, or wholly prevented, according to the quantity of sugar present.

If the quantity added does not exceed two grains and a half to the ounce, the crystallization is not prevented; but even this small quantity is perceptible by a degree of blackness that appears after evaporation: occasioned, as I suppose, by the action of a small excess of acid on the sugar.

If five grains have been added, the crystallization is very imperfect, and soon disappears in a moist air by deliquescence of the sugar. The blackness is also deeper than in the former case.

By the addition of ten grains to the ounce, the crystallization of the salts is entirely prevented, and the degree of blackness, and disposition to deliquesce are of course more manifest than with smaller quantities.

As I was aware that the sugar obtained from diabetic urine is a different substance from common sugar (approaching more nearly to the sugar of figs), I had the precaution to repeat the same series of experiments upon serum, to which I made corresponding additions of dry sugar, that I had formerly extracted from the urine of a person who voided it in considerable quantity; and I found the effects to be perfectly similar in every respect.

As a further test of the absence or presence of sugar, I found it convenient to add a little nitric acid to the salts that remained after crystallization of the drop. If the serum has been

* I presumed that this portion of albumen was retained in solution by the alkali redundant in serum, and added the acid for the purpose of neutralizing it.

successfully coagulated without any addition of sugar, the addition of nitric acid merely converts the muriatic salts into nitrates, and nitrate of soda is seen to crystallize without foam or blackness. But when sugar has been added, a white foam rises round the margin of the drop, and if further heat be applied, it becomes black in proportion to the quantity of sugar present.

Such are the appearances when the proportions have been duly adjusted, and the proper heat for coagulation applied. I must own, however, that I could not always succeed to my satisfaction at the time when these experiments were conducted, and I am inclined to ascribe occasional failures to having used more muriatic acid than was really necessary, which by excess of heat might redissolve part of the coagulated albumen, and thence occasion appearances, which, without careful discrimination, might be ascribed to sugar.

After having, by this course of experiment, satisfied myself as to the phenomena exhibited by serum in its natural state, and the effects of any small additions of sugar, I then proceeded to the examination of such specimens of diabetic blood or of serum, as I was able to procure.

The first which I examined was a portion of blood that had been taken from a person whose urine had been analysed, and found to contain sugar. This blood had been dried, when fresh, by a gentle heat, so as not to coagulate the serum. After being reduced to powder, it was mixed with water, in order that every thing which remained soluble might be extracted. A little muriatic acid was then added, and sufficient heat applied for coagulation of the albumen. The water that separated after coagulation was found to contain the salts of the blood, but no trace whatever of sugar.

A second specimen of dried blood, that had been ascertained to be diabetic on the same evidence as the preceding, was examined in a similar manner, with the same result, as no appearance of sugar could be discerned.

In a third instance, I had some serum from the blood of a person whose urine had been tasted, and found "*very sweet.*" (I had no opportunity of procuring any of this urine for analysis). After a portion of this serum had been coagulated, with the addition of the usual proportion of muriatic acid, there was no appearance whatever of sugar. But when three grains of diabetic sugar had been added to another ounce of the same serum, the presence of this quantity was manifest by the same process.

I had also a fourth opportunity of examining serum of a person whose urine contained so much saccharine matter, that an ounce of it yielded, by evaporation, thirty-six grains of extract.

extract. In this instance I was not so successful in my experiment; for, though I was satisfied that no sugar was present, there certainly was a degree of blackness, which might have been occasioned by about one grain and a half of sugar in the ounce of serum. But this black matter appeared not to be sugar: it was more easily dried than sugar: it was not fusible by heat as sugar is: and its refractive power* was too great for that of sugar.

I unfortunately had no opportunity of repeating the experiment on a second portion of the same serum, having inconsiderately employed it for other experiments, and coagulated it at the same time with the former.

In the next experiment I added half a dram of the urine of the same person to six drams of the serum, and with a due proportion of diluted muriatic acid coagulated as before. Although the quantity of extract added did not exceed $\frac{1}{16}$, or two grains and a quarter of extract, the difference was very manifest by the darkness of the colour and the defective crystallization of the salts.

To the remaining quantity of the serum I had added twice the former proportion of the urine, and found that this quantity did not wholly prevent the crystallization of the salts during the evaporation of the drop.

The result of these trials was such, as to satisfy me that the serum in this instance contained no perceptible quantity of sugar, or at least that the water separable from the coagulated serum did not contain one-thirtieth part of that proportion which I had found in the urine of the same person.

In order to account for the presence of sugar in the urine, we must consequently either suppose a power in the kidneys of forming this new product by secretion, which does not seem to accord with the proper office of that organ; or, if we suppose the sugar to be formed in the stomach by a process of imperfect assimilation, we must then admit the existence of some channel of conveyance from the stomach to the bladder, without passing through the general system of blood-vessels. That some such channel does exist, Dr. Darwin † endeavoured to ascertain, by giving large doses of nitre, which he could perceive to pass with the urine, but could not detect in its passage through the blood; and he imagined the channel by which it was conveyed to be the absorbent system, upon the

---

* The method by which this was tried has since that time been described in the Philosophical Transactions for 1802.

† Account of the retrograde Motion of the absorbent Vessels, by Charles Darwin.

 supposition supposition that they might admit of a retrograde motion of their contents.

Without adopting the theory of Dr. Darwin, it did appear to me that the fact deserved to be ascertained by some test more decisive than nitre, and I conceived that if prussiate of potash could be taken with safety, its presence would be discerned by means of a solution of iron in as small proportion as almost any known chemical test. Upon trial of this salt, I found that a solution of it might be taken without the least inconvenience, and that in less than one hour and a half the urine became perceptibly impregnated, and continued so to the fifth or sixth hour, although the quantity taken had not amounted to more than three grains of the salt.

After a few previous trials of the period when the principal impregnation of the urine might be expected, and when the presence of the prussiate (if it existed in the blood) might with most reason be presumed to occur, a healthy person about thirty-four years of age was induced to take a dose corresponding to three grains and a half of the dry salt, and to repeat it every hour to the third time. The urine being examined every half hour, was found in two hours to be tinged, and to afford a deep blue at the end of four hours. Blood was then taken from the arm, and the coagulum, after it had formed, was allowed to contract, so that the serum might be fully separated. The presence of the prussiate was then endeavoured to be discovered by means of a solution of iron, but without effect; and as I thought that the redundant alkali (which had been ascertained to prevail in this serum) might tend to prevent the appearance of the precipitate, I added a small quantity of dilute acid; but still I could not discern that any degree of blueness was occasioned by it.

This experiment having been repeated a second time with the same result, seemed to me nearly conclusive with respect to the existemce of some passage, by which substances certainly known to be in the stomach may find their way to the bladder without being mixed with the general mass of circulating fluids.

Being desirous of ascertaining whether the prussiate could be discovered in any other secretions, I have repeatedly examined my saliva, at times when the urine has manifested a very strong blue, by adding solution of iron, but I could at no time perceive the saliva to be tinged.

I have also, during a severe cold, accompanied with profuse running of water from the nose, made a similar examination of this discharge, but have not been able to perceive any trace of the prussic acid.

It

It was nearly in this state that I left the inquiry at the period I have mentioned, and I do not remember to have made any other experiments, when I requested your assistance in making trial of the serum that is secreted in consequence of the application of a blister. Your report upon the result of your experiments, in addition to those which I have above related, nearly satisfied me as to the existence of some unknown channel of conveyance by which substances may reach the bladder.

With respect to Dr. Darwin's conception of a retrograde action of the absorbents, it is so strongly opposed by the known structure of that system of vessels, that I believe few persons will admit it to be in any degree probable.

Since we have become acquainted with the surprising chemical effects of the lowest states of electricity, I have been inclined to hope that we might from that source derive some explanation of such phenomena. But though I have referred* secretion in general to the agency of the electric power with which the nerves appear to be indued, and am thereby reconciled to the secretion of acid urine, from blood that is known to be alkaline, which before that time seemed highly paradoxical; and although the transfer of the prussiate of potash, of sugar, or of other substances, may equally be affected by the same power as acting cause, still the channel through which they are conveyed remains to be discovered by direct experiment.

I have, indeed, conjectured that, by examining the blood in the abdominal vessels, or contents of the lacteals, it might be possible to detect them *in transitu;* but I have not been inclined to make such experiments on living animals, as would perhaps throw light upon the subject.

W. H. WOLLASTON.

*January* 1, 1811.

---

### *Reply of Dr.* Marcet *on the same Subject.*

*Russel Square, January* 8, 1811.

I WAS anxious that the specious hypothesis of the presence of sugar in diabetic blood, which had been sanctioned by the authority of Dr. Rollo and Mr. Cruickshank, and which I had myself urged in support of their theory, fourteen years ago,

* Philosophical Magazine for June, 1809.

ago, in an inaugural publication, should no longer obtain an undue weight amongst physiological inquirers.

With regard to the experiments which I tried at your request some years ago, with a view to ascertain whether prussiat of potash taken into the stomach, and found to exist in the urine, could also be detected in other secretions, I find, on referring to my memorandums, the following particulars, which I shall transcribe verbatim.

"August 19, 1807. Having heard from Dr. Wollaston, that prussiat of potash could be taken into the stomach with perfect safety, and that its presence could afterwards be discovered in the urine, but not in the serum; and being invited by him to follow up this inquiry, with a view to connect it with the theory of diabetes, I tried the following experiments.

*Experiment* 1.

"After having satisfied myself, by trials made by some medical gentlemen upon themselves, that considerable doses of prussiat of potash might be taken without the least inconvenience, I gave to a young woman labouring under diabetes mellitus, five grains of prussiat of potash dissolved in water, and this was repeated every hour till she had taken thirteen or fourteen such doses. After the fifth dose, her urine, by the addition of a drop or two of a solution of sulphat of iron, turned blue instantly. At this period of the experiment, a blister was applied to her stomach, and after a few hours, whilst still taking the prussiat of potash, and whilst the urine strongly indicated its presence, the blister was cut and the serum collected. This serous fluid being, in the same manner as the urine, subjected to the action of a solution of sulphat of iron, did not suffer any change of colour in the least indicative of the presence of prussic acid. Yet the urine still remained capable of imparting a blue colour to solution of iron, fifteen hours after taking the last dose of the prussiat of of potash.

*Experiment* 2.

"The same person being soon afterwards put upon a course of ferruginous medicines, and having taken considerable quantities of sulphat of iron, an idea naturally occurred to me that the phenomenon might perhaps be reversed; but upon adding prussiat of potash to the urine, no vestige of iron could be discovered, and the same attempt was repeated several times with the same negative result.

*Experiment* 3.

"Dec. 2, 1807. The fluid obtained by means of a blister (as in Experiment 1), being not immediately derived from the the circulation, since it may be considered as the product of a secretion, I was desirous of repeating Dr. Wollaston's experiment on the serum itself, under circumstances of impregnation similar to those in which the serum of the blister was examined.

"For this purpose, a young woman after taking, in divided doses, about a dram of prussiat of potash in the course of twelve hours, lost some blood by cupping, an operation which had been ordered for a local complaint under which she laboured. The serum having been allowed to separate, and a little nitric acid having been added to it, not the least vestige of prussic acid appeared in applying the test of sulphat of iron, although the urine made during the six hours which preceded and followed the cupping, was strongly impregnated with that acid, and struck a vivid blue upon adding the smallest quantity of iron."

I have only to observe, in addition to these particulars, that the susceptibility with which prussiat of potash is transmitted to the bladder, seems to vary in different individuals; for in five trials, made at Guy's Hospital, in Nov. 1805, I failed of discovering any vestige of that salt in the urine of persons who had taken it in quantities sufficient to produce its appearance in others. Three of these individuals, I should observe, were at the time under mercurial treatment; and an idea occurred to me that mercury having a great affinity for prussic acid, the presence of that metal in the system might prevent the effect in question. But as in the two other failures, no mercury was present, I cannot lay any stress upon that conjecture. It may be proper to mention, that in the frequent trials which I have made with the prussiat of potash, no symptom or inconvenience whatever has ever occurred which could be ascribed to that salt.

ALEX. MARCET.

P. S. Whilst revising the proof of this sheet, it has been observed to me by some friends and in particular by Dr. Henry of Manchester, and Dr. R. Pearson of London, that in order to show distinctly that certain substances find their way to the bladder, without passing through the general circulation, it would be necessary to examine the arterial, as well as the venous blood, since it is not impossible that the whole of the sugar in diabetes, or the prussiat of potash in the experiments above related, may be conveyed to the urinary organs by the arteries, without entering the venous system. According to this hypothesis, it may be conceived that the same substances when conveyed by the arteries to distant parts of the body, may return by the absorbent system, and might in that that case be discovered in the thoracic duct. This view of the subject may deserve further investigation; and I hope that this curious question will soon be decided by appropriate experiments.

